# Role of Gated Myocardial Glucose Metabolic Imaging in Assessing Left Ventricular Systolic Dyssynchrony after Myocardial Infarction and the Influential Factors

**DOI:** 10.1038/s41598-018-29636-8

**Published:** 2018-07-25

**Authors:** Xiaoliang Shao, Jianfeng Wang, Yi Tian, Shengdeng Fan, Feifei Zhang, Wei Yang, Wenchong Xin, Yuetao Wang

**Affiliations:** 1 Department of Nuclear Medicine, The Third Affiliated Hospital of Soochow University, Changzhou, 213003 China; 2 Department of Nuclear Medicine, Beijing Anzhen Hospital, Capital Medical University, Beijing, 100029 China; 3 Department of Anesthesiology, The Third Affiliated Hospital of Soochow University, Changzhou, 213003 China

## Abstract

In this study, we investigated the role of gated myocardial glucose metabolic imaging in assessing left ventricular (LV) systolic dyssynchrony after myocardial infarction (MI) and explored the influencing factors. Bama mini-pigs were divided into normal group and MI group and subjected to gated myocardial metabolic imaging (GMMI) and gated myocardial perfusion imaging (GMPI). The phase bandwidth (BW), standard deviation (SD) and the latest activation site of left ventricle were obtained using program Cedars QGS. The results showed that (1) BW and SD obtained in GMMI and GMPI showed significant correlation in pigs with MI, but not in the normal pigs, (2) BW and SD obtained in GMMI and GMPI had good consistency in both normal pigs and MI pigs, (3) GMMI and GMPI had a 66.7% identity in determining the latest activation site of left ventricle in the normal pigs and 77.8% identity in determining the latest activation site of left ventricle in pigs with MI. Multivariate stepwise regression analysis showed that total perfusion deficit and summed motion score were independent factors affecting BW and SD in GMMI. In conclusion, phase analysis of GMMI images could objectively reflect LV systolic dyssynchrony resulted from interactions of multiple factors.

## Introduction

Cardiac resynchronization therapy (CRT) is an effective treatment for severe heart failure. But according to the existing guidelines^1^, nearly 30% of heart failure patients do not respond to CRT favorably^2^. Series of studies showed that location of myocardial scars^3,4^, amount of viable myocardium^5^, left ventricular (LV) motion patterns^6^ and LV systolic synchrony^7^ were important factors affecting patients’ response to CRT.

Theoretically, techniques that could be used to simultaneously assess scar burden and location, myocardial viability, LV function and systolic dyssynchrony will help guide the placement of CRT electrodes and solve non-response problems. Echocardiography is non-invasive, cost-effective and easy to access, but there were significant inter-observer (4–24%) and intra-observer variations (7–72%) among many parameters^8^. Cardiovascular magnetic resonance has been used to assess LV myocardial scar and systolic dyssynchrony, but it is time-consuming and involves significant user interaction^9^. Gated myocardial perfusion imaging (GMPI) is widely accepted, and a single resting GMPI scan can simultaneously assess the global and regional functions, scar burden and location, systolic synchrony and latest activation site of left ventricle. Zhou *et al*.^10^ applied GMPI to guide the placement of CRT electrodes and obtained good reproducibility and high response rate. GMPI phase analysis now has become the standard reference for assessing LV systolic dyssynchrony^11^. Compared with GMPI, ^18^F-fluorodeoxyglucose (FDG) gated myocardial metabolic imaging (GMMI) has higher spatial resolution and is more accurate for assessing extent of scar and myocardial viability^12,13^. Pilot studies^14^ indicated that clinical non-responders to CRT could benefit from assessment of ^18^F-FDG positron emission tomography (PET)/computed tomography (CT). However, very few clinical investigations compared phase analyses of these two methods^15,16^ and no consistent conclusions were reached possibly due to relative confounding factors of these retrospective studies. Therefore, in this study we strictly controlled the influencing factors and used GMPI phase analysis as the reference standard to evaluate the feasibility of GMMI phase analysis for clinical utilization. We also analyzed the influencing factors for improving CRT responsiveness of heart failure patients.

## Results

### Phase analysis and influential factors of GMMI images of pigs in normal group

The heart rate and QRS duration of the 9 pigs in the normal group were 72.8 ± 23.3 bpm and 61.0 ± 7.8 ms, respectively. Phase analysis of their GMMI images showed that LV end-diastolic volume (EDV) was 46.3 ± 8.3 ml, end-systolic volume (ESV) was 9.6 ± 5.6 ml and LV ejection fraction (LVEF) was 80.1 ± 8.2%.

**Figure 1:**
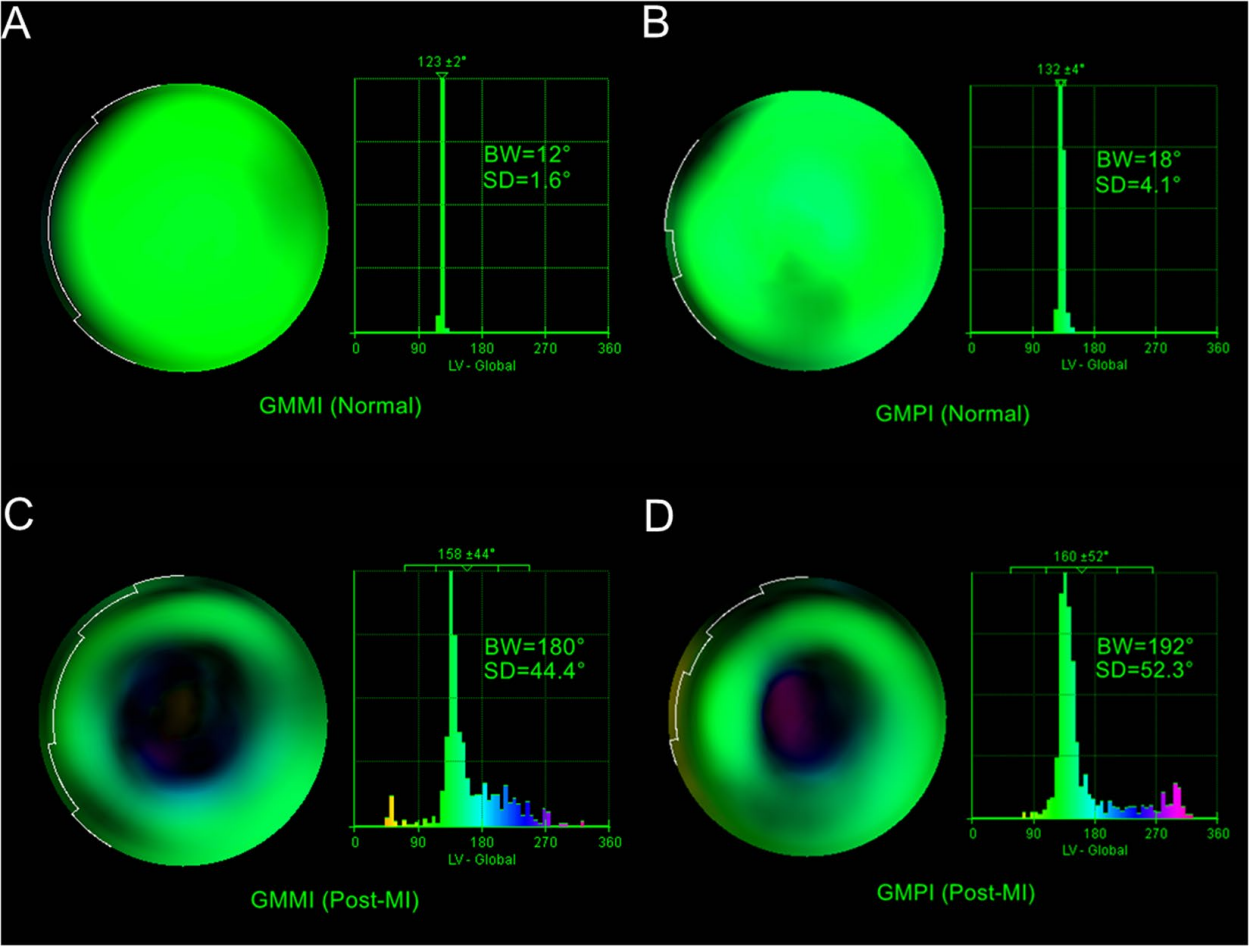
Bull’s eye plots and phase histograms obtained from GMMI and GMPI phase analysis. (**A**) GMMI phase analysis of a normal pig. (**B**) GMPI phase analysis of the same normal pig. (**A**,**B**) show a uniform color-scale distribution in the bull’s eye plot and a narrow and sharp peak with two sides being symmetrical in the phase histogram. (**C**) GMMI phase analysis of a pig with MI. (**D**) GMPI phase analysis of the same pig with MI. (**C**,**D)** show an inhomogeneous color-scale distribution in the bull’s eye plot and an evidently widened and asymmetrical peak in the phase histogram.

**Figure 2:**
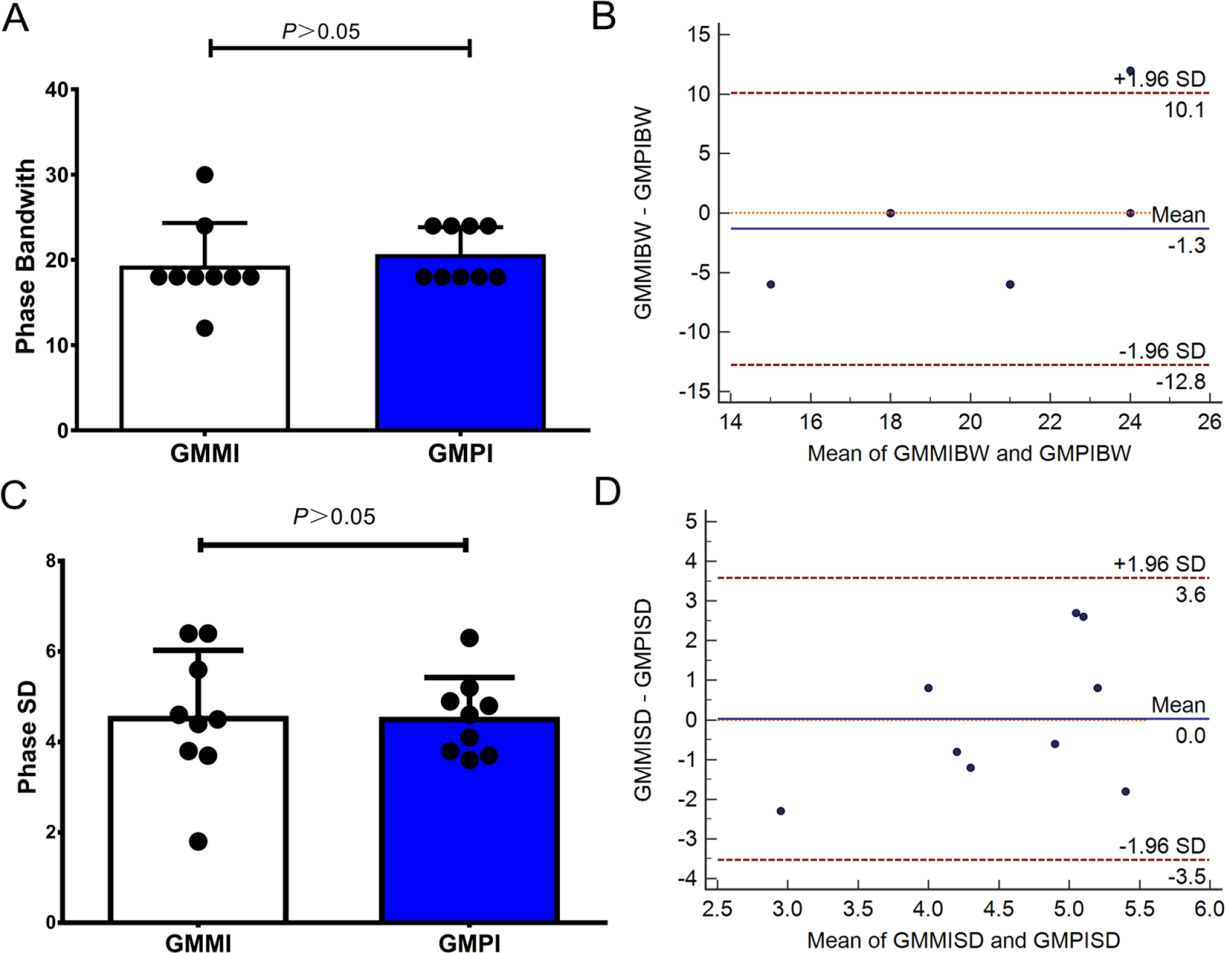
Comparison in phase analysis between GMMI and GMPI methods for pigs in the normal group. (**A**) Phase BW obtained from GMMI, showing no difference from that obtained from GMPI (*P* > 0.05). (**B**) Bland-Altman analysis, showing good consistency in BW obtained using the two methods. Note: there are 9 data points, but some data are completely overlapped, making it look like 5 plots. (**C**) Phase SD obtained from GMMI, showing no differences from that obtained from GMPI (*P* > 0.05). (**D**) Bland-Altman analysis, showing good consistency in SD obtained using the two methods.

Phase analysis of pigs in the normal group showed a uniform color-scale distribution in the bull’s eye plot and a narrow and sharp peak with two sides being symmetrical in the phase histogram obtained from either GMMI or GMPI, as shown in Fig. 1A,B, indicating good synchrony. The bandwidth (BW) and standard deviation (SD) of GMMI were 19.3 ± 5.0° and 4.6 ± 1.4°, showing no differences from those of 20.7 ± 3.2° and 4.6 ± 0.9° obtained from GMPI (*P* > 0.05), respectively, as shown in Fig. 2A,C. In addition, the normal values of BW and SD obtained from both imaging methods had no significant correlation (*P* > 0.05 for all) as shown with Pearson’s correlation coefficients r_BW_ = 0.03 and r_SD_ = −0.17. Bland-Altman analysis showed that BW and SD values obtained from GMMI were in rough agreement with those obtained from GMPI with differences of −1.33 ± 5.83° and 0.02 ± 1.81° for BW and SD, respectively, indicating no significant difference from the value 0 (*P* > 0.05). The 95% limits of agreement were −12.76° to 10.10° for BW and −3.53° to 3.57° for SD, as shown in Fig. 2B,D.

The latest activation site of left ventricle in GMMI was 55.6% (5/9) in the anterior, 22.2% (2/9) in the anteroseptal, 11.1% (1/9) in the inferoseptal and 11.1% (1/9) in the anterolateral wall. The latest activation site of left ventricle in GMPI was 44.4% (4/9) in the anterior, 22.2% (2/9) in the anteroseptal, 22.2% (2/9) in the inferoseptal and 11.1% (1/9) in the inferior wall, showing an agreement of 66.7% (6/9).

The analysis of possible factors affecting LV systolic dyssynchrony in GMMI showed that (1) BW and SD had a significant negative correlation to the ratio (SUVm/b) of standard uptake values of myocardium (SUVmyo) and mediastinal blood pool (SUVbp) (*P* < 0.05 for all), and (2) SD had a significant negative correlation to LVEF (*P* < 0.05), but not EDV and ESV (*P* > 0.05 for both), as shown in Table 1.

**Table 1:** Factors affecting LV systolic synchrony in GMMI for pigs in normal group.

Parameter	BW		SD	
	r	*P*	r	*P*
QRS duration	0.41	0.308	0.56	0.148
EDV	0.62	0.074	0.26	0.496
ESV	0.67	0.051	0.57	0.108
LVEF	−0.61	0.083	−0.69	0.041*
SUVm/b	−0.77	0.016*	−0.89	0.001*

EDV: end-diastolic volume; ESV: end-systolic volume; LVEF: left ventricular ejection fraction; SUVm/b: ratio of standard uptake values of myocardium and mediastinal blood pool.

*Indicates significant statistical difference.

### Phase analysis and influential factors of GMMI images of pigs in myocardial infarction (MI) group

**Figure 3:**
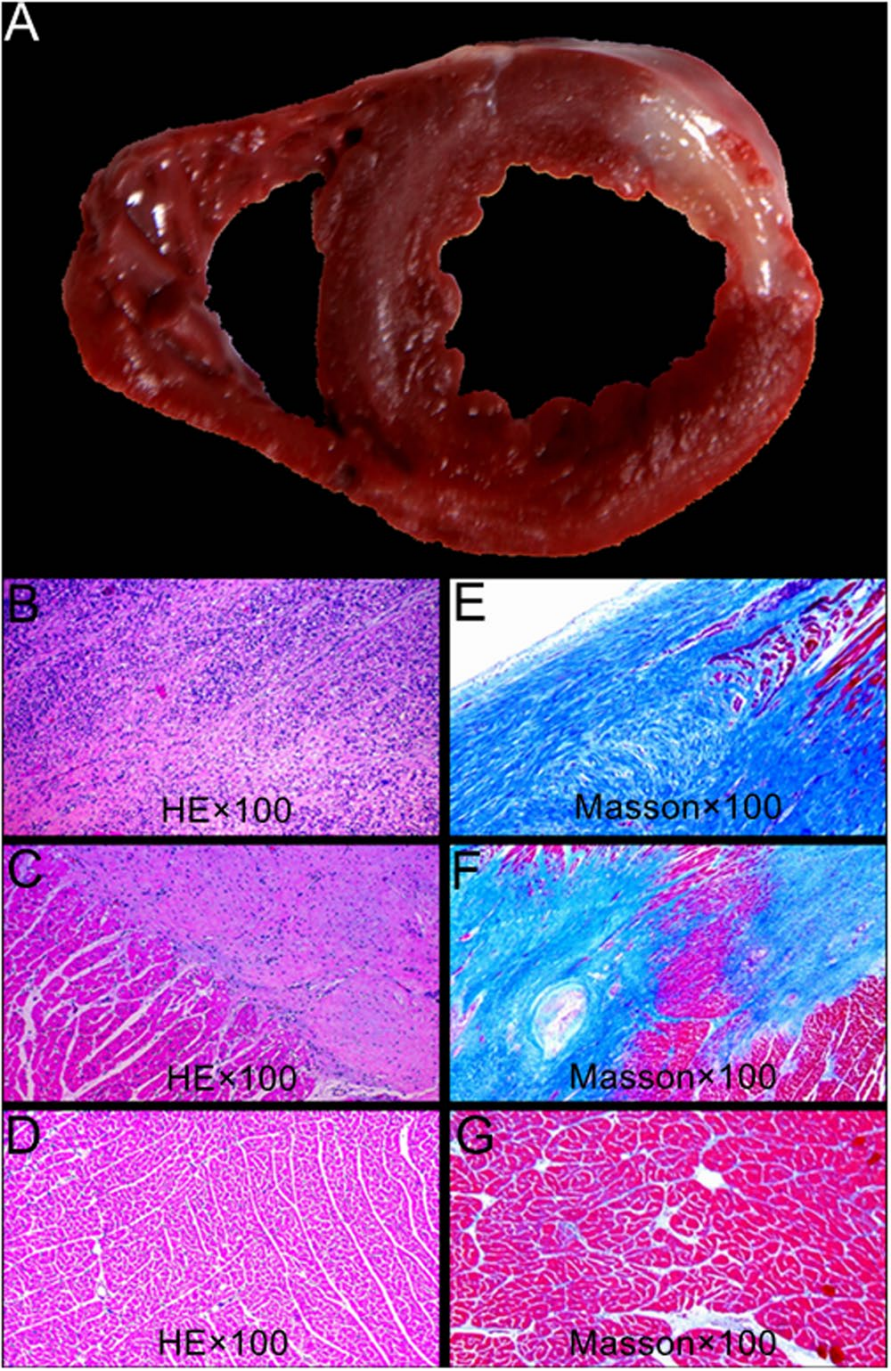
Pathological and histopathological images of left ventricle of pigs in MI group after HE and Masson staining. (**A**) Infarcted myocardium appears in white while normal myocardium appears in red in TTC stained slides. (**B**,**E**) HE and Masson staining of infarcted segment, showing full of necrotic myocardium, obvious of myocardium fibrosis, and few viable myocardium. (**C**,**F**) HE and Masson staining of infarct margin segment, showing coexistence of necrotic and viable myocardium. (**D**,**G**) HE and Masson staining of normal myocardium.

MI was successfully established in the 9 pigs in the MI group. No pig died till 30 to 35 days after modeling. The heart rate of pigs with MI was 72.1 ± 10.1 bpm and QRS duration was 83.3 ± 47.7 ms. The total perfusion deficit (TPD) of left ventricle caused by modeling was up to 43.9 ± 14.0%, mainly present in the blood-supplying myocardium of left anterior descending (LAD) artery, and the level of troponin I was 267.88 ± 86.96 ng/ml. LV EDV and ESV of pigs with MI was 54.4 ± 17.1 ml and 16.9 ± 16.6 ml, respectively. The LV cavity of pigs with MI was significantly greater than that of the normal group (*P* < 0.05); LVEF of pigs with MI was 53.2 ± 16.6%, which was significantly less than that of normal group (*P* < 0.05). The summed motion score (SMS) of pigs with MI was 23.2 ± 13.9, summed thickening score (STS) was 13.6 ± 12.0, the total scar score was 9.4 ± 4.7, and the number of viable myocardial segments was 10 ± 4, respectively. The general pathological examination of left ventricle of pigs with MI showed slightly enlarged LV cavity, slightly thinned infiltration area and no thrombosis formation. Both Hematoxylin-Eosin (HE) and Masson staining showed infarcted fibrous tissue and a small amount of viable myocardium in the infarcted area, coexistence of infarcted fibrous tissue and viable myocardial tissue in the infarcted area margin, and normal myocardial tissue in the distal end, as shown in Fig. 3A–G.

**Figure 4:**
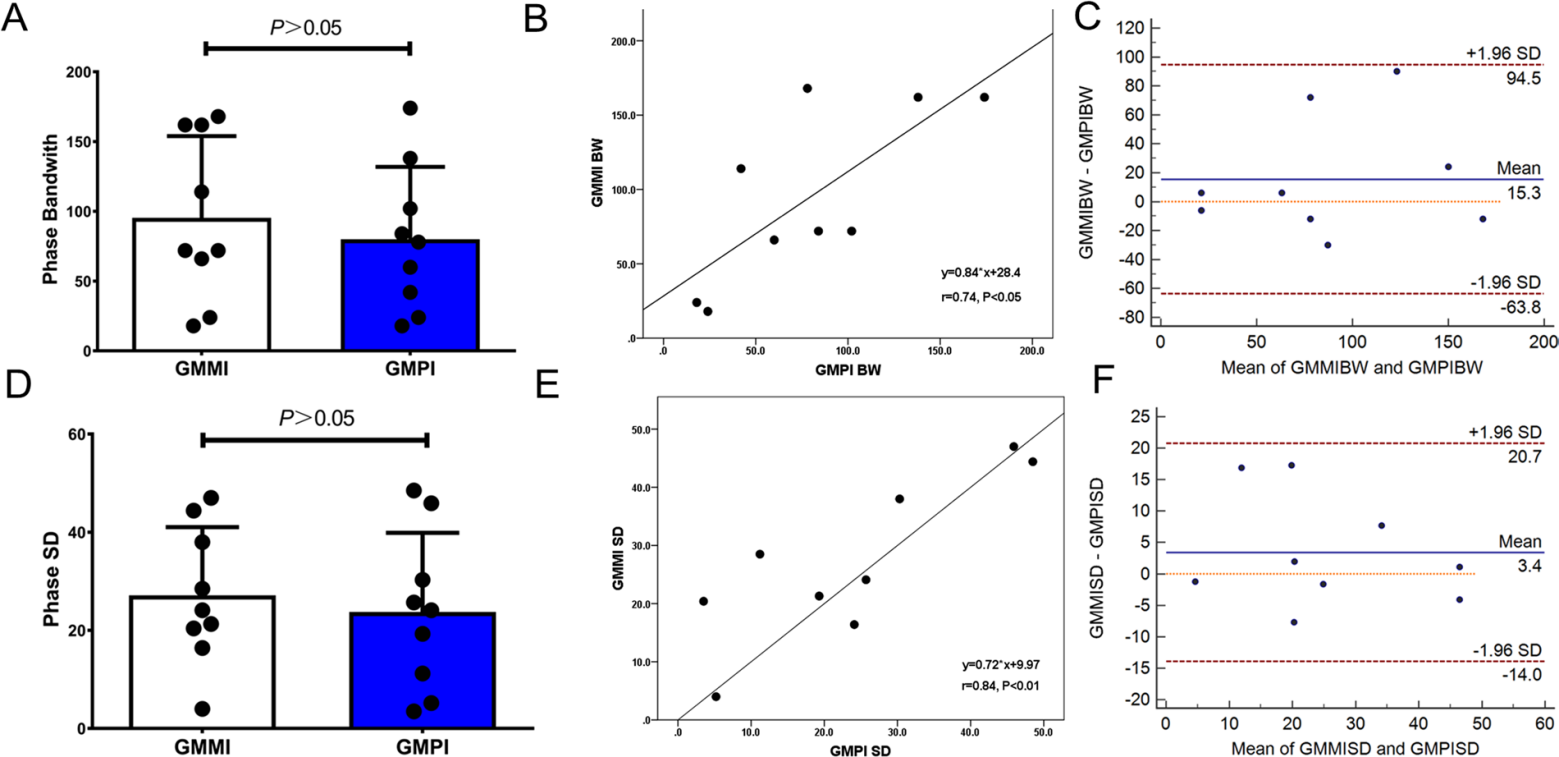
Comparison in phase analysis between GMMI and GMPI methods for pigs in the MI group. (**A**) Phase BW obtained from GMMI, showing no differences from that obtained from GMPI (*P* > 0.05). (**B**) Pearson linear correlation analysis, showing a significant and positive correlation between GMMI and GMPI for BW (r_BW_ = 0.74, *P* < 0.05). (**C**) Bland-Altman analysis, showing good consistency in BW obtained using the two methods. (**D**) Phase SD obtained from GMMI, showing no difference from that obtained from GMPI (*P* > 0.05). (**E**) Pearson linear correlation analysis, showing a significant and positive correlation between GMMI and GMPI for SD (r_SD_ = 0.84, *P* < 0.05); (**F**) Bland-Altman analysis, showing good consistency in SD obtained using the two methods.

Phase analysis of pigs with MI showed an inhomogeneous color-scale distribution in the bull’s eye plot and an evidently widened and asymmetrical peak in the phase histogram, as shown in Fig. 1C,D, clearly indicating the presence of LV systolic dyssynchrony. Phase analysis of GMMI images showed BW and SD of pigs with MI were 95.3 ± 58.7° and 27.1 ± 13.9°, respectively, significantly higher than those of the normal group (*P* < 0.05), indicating significant intra-LV systolic dyssynchrony after MI, but showing no significant differences from BW of 80.0 ± 51.9° and SD of 23.7 ± 16.1° obtained using GMPI (*P* > 0.05 for all), as shown in Fig. 4A,D. There was a significant positive correlation between the values obtained by these two imaging methods based on the Pearson’s correlation coefficients of r_BW_ = 0.74 and r_SD_ = 0.84 (*P* < 0.05 for all), as shown in Fig. 4B,E and high reliability of inherent consistency (intraclass correlation coefficient for BW: 0.85, 95% CI: 0.32–0.97 and intraclass correlation coefficient for SD: 0.91, 95% CI: 0.58–0.98, *P* < 0.05 for both). Bland-Altman analysis showed that the values obtained with GMMI phase analysis were slightly higher than those with GMPI one, with difference of 15.33 ± 40.37° in BW and 3.38 ± 8.85° in SD, respectively, but there was no significant difference compared to the value 0 (*P* > 0.05) and the 95% limits of agreement were −63.80° to 94.46° for BW and −13.97° to 20.72° for SD, as shown in Fig. 4C,F.

GMMI showed that the latest activation sites of left ventricle counted for 44.4% (4/9) in apical region, 22.2% (2/9) in the anterior, 22.2% (2/9) in the inferolateral and 11.1% (1/9) in the inferoseptal wall, while GMPI showed that the latest activation sites of left ventricle counted for 55.6% (5/9) in apical region, 22.2% (2/9) in the inferior, 11.1% (1/9) in the inferoseptal and 11.1% (1/9) in the anterolateral wall, showing 77.8% (7/9) of agreement.

Univariate analysis of possible factors affecting post-MI LV systolic dyssynchrony showed that (1) BW in GMMI had significant, positive correlations with TPD, ESV, SMS and total scar score, but negative correlations with LVEF and the number of viable myocardial segments; and (2) SD in GMMI had significant, positive correlations with TPD, ESV, SMS and STS, but significant, negative correlations with LVEF and number of viable myocardial segments, as listed in Table 2. Furthermore, multivariate stepwise regression analysis showed that TPD was an independent factor affecting BW in GMMI (*R*^2^ = 0.59, *F* = 12.63, *P* = 0.009), whereas SMS was an independent factor affecting SD in GMMI (*R*^2^ = 0.57, *F* = 11.74, *P* = 0.011).

**Table 2:** Factors affecting LV systolic synchrony in GMMI for pigs in MI group.

Parameter	BW		SD	
	r	*P*	r	*P*
QRS duration	−0.49	0.183	−0.61	0.081
TPD	0.80	0.009*	0.73	0.027*
EDV	0.58	0.105	0.62	0.077
ESV	0.67	0.047*	0.78	0.014*
LVEF	−0.68	0.045*	−0.78	0.013*
SMS	0.72	0.030*	0.79	0.011*
STS	0.62	0.076	0.71	0.031*
Scar score	0.71	0.031*	0.66	0.052
Viable segments	−0.75	0.020*	−0.73	0.025*
SUVm/b	−0.18	0.635	−0.24	0.544

TPD: total perfusion deficit; EDV: end-diastolic volume; ESV: end-systolic volume; LVEF: left ventricular ejection fraction; SMS: summed motion score; STS: summed thickening score; SUVm/b: ratio of standard uptake values of myocardium and mediastinal blood pool.

*Indicates significant statistical difference.

## Discussion

### Accuracy of quantitatively determining BW and SD using GMMI phase analysis

Whether GMMI phase analysis is clinically feasible depends on its accuracy for quantitative determination of BW and SD values. In this study, we performed animal experiments and compared the consistency and linear correlation of BW and SD obtained by using GMMI phase analysis with those by GMPI one. The results showed that for normal group, BW and SD values obtained by GMMI and GMPI phase analyses were in a good agreement, but had no significant correlation, possibly due to that there was a narrow and sharp peak in phase histogram and narrow normal ranges of BW and SD of the normal group. Thus, under the time resolution (5.6°)^17^, it is impossible to demonstrate their correlation. For the MI group, BW and SD values obtained by GMMI and GMPI phase analysis were in a good agreement and had a significant correlation (r_BW_ = 0.74 and r_SD_ = 0.84). Furthermore, for both normal and MI groups, BW and SD values obtained through GMMI phase analysis were not overestimated or underestimated. In previous studies comparing GMMI and GMPI phase analyses^15,16^, all enrolled patients had coronary heart disease. However, due to differences in the accompanied diseases, degrees of LV remodeling and cardiac function impairment, as well as image qualities and reconstruction methods, the correlations of BW and SD values obtained from GMMI and GMPI phase analyses were different (Pazhenkottil *et al*.^16^: r_BW_ = 0.88 and r_SD_ = 0.88 vs. Wang *et al*.^15^: r_BW_ = 0.6 and r_SD_ = 0.58). Compared with these studies, we mainly utilized mini-pigs, which have been shown to have a better translational bridge between preclinical and clinical studies compared with other animals^18^, to simulate the clinical situations of patients with MI not accompanied with other diseases (diabetes, hypertension, valvular disease, etc.) and assessed the accuracy of GMMI phase analysis technique.

### Determination of the latest activation sites of left ventricle

Implantation of CRT electrodes in the latest activation sites of left ventricle can rectify LV systolic dyssynchrony to the greatest degree. Khan^19^ found that placement of the LV lead to the latest sites of contraction and away from the scar could significantly reduce mortality of patients with heart failure and heart-failure-related hospitalization rate. Therefore, accurate assessment of the latest activation sites is particularly necessary. In this study, we showed that GMMI phase analysis had slightly higher consistency with GMPI one in determination of the latest activation sites of left ventricle in MI group than in normal group (77.8% vs 66.7%), roughly consistent with that of Wang *et al*.^15^. In addition, we also found that the latest activation sites were located in LAD artery distribution area in 88.9% of pigs with MI using GMMI phase analysis, while in 66.7% of pigs with MI using GMPI phase analysis. The underlying mechanism may be related to that the occlusion-induced MI of LAD artery distribution could lead to loss of metabolic activity of cardiomyocytes and weakening/disappearance of local myocardial systolic function, presenting as delayed mechanical contraction.

### Risk factors for LV systolic dyssynchrony in GMMI

The results showed that BW and SD of the normal group, but not the MI group, in the GMMI were significantly and negatively correlated with SUVm/b. Thus, it is clear that the quality of GMMI images is related to the accuracy of phase analysis. When the quality of images is poor, the ratio of SUV values of myocardial and mediastinal blood pools is low, indicating that there are risks of overestimating BW and SD.

This study showed that both BW and SD in the MI group were significantly and positively correlated with TPD of left ventricle, but evidently and negatively correlated with LVEF. Multivariate linear regression analysis showed that TPD was an independent factor affecting BW in GMMI. After MI, the greater the extent and degree of myocardial perfusion defects are, the more obvious the LV systolic dyssynchrony is and the more severe the damage to LV systolic function is. HF-Action study^20^ analyzed resting GMPI images of 2331 patients with heart failure and found that SD was significantly and positively correlated with summed rest score (r = 0.66, *P* < 0.0001), but negatively correlated with LVEF (r = −0.50, *P* < 0.0001), being consistent with our findings. Our study also found that after MI, the higher the total scar score is, the fewer the viable myocardial segments are and the more obvious the LV systolic dyssynchrony is. CRT can ameliorate LV systolic dyssynchrony. But too great myocardial scar burden can also cause nonresponse to CRT. Adelstein *et al*.^3^ studied patients with ischemic cardiomyopathy and found that when LV summed rest score ≥27, the improvement of post-CRT LV function was not obvious and the prognosis of patients was poor. Ypenburg *et al*.^21^ found that the sensitivity and specificity of predicting clinical response to CRT with the viable myocardial segments ≥11 were 74% and 87%, respectively.

This study also found that after MI, (1) BW was significantly and positively correlated with ESV and SMS, (2) SD was obviously associated with ESV, SMS and STS of left ventricle, and (3) SMS was an independent factor affecting SD in GMMI. These results indicate that post-MI LV systolic dyssynchrony is related not only to global systolic function but also to ESV as well as regional systolic function decline (SMS, STS). Although the factors affecting BW and SD in GMMI phase are not exactly the same, both of them can be used to determine the presence of LV systolic dyssynchrony and assess the prognosis of patients with MI^22^.

### Clinical applications

Although GMPI has been widely accepted to assess LV systolic dyssynchrony automatically and reproducibly, it still faces several challenges, such as limited temporal resolution as well as noise and artifact from areas with significantly reduced perfusion due to scar^23^. Because of higher spatial resolution and accuracy for assessing myocardial viability (hypoperfusion in GMPI, normal metabolism in GMMI), PET GMMI detects LV contour more accurately than GMPI, which has emerged as a suitable modality to study the metabolic mechanisms of LV systolic dyssynchrony and the effects of CRT. PET/CT is not cost effective. But if this non-invasive imaging technology can significantly improve CRT response rate, it would be still acceptable, which needs further verification of multicenter clinical trials. Our findings may provide some evidences for development of such clinical trials.

### Limitations

First of all, the study is not a self-control study because repeated anesthesia can easily affect cardiac function and potentially lead to reduction of myocardial glucose metabolism of experimental pigs^24^. Second, the experimental animals used in this study were Guangxi Bama mini-pigs with a heart smaller than that of an adult. Thus, there is partial volume effect in the imaging process, especially in GMPI. Although 128 × 128 matrix, 1.45-fold amplification and an increased acquisition time were used in imaging, there was still possibility of overestimating cardiac function. Nevertheless, a previous study^18^ showed the experimental data on mini-pigs were more similar to those of humans. Third, many factors could affect myocardial substrate metabolism and myocardial glucose metabolism varies greatly. Although hyperinsulinemic -euglycemic clamp could promote glucose uptake in myocardia to the maximum degree and help obtain images with excellent quality, it requires longer operative time under anesthesia, which may affect cardiac function of the experimental animals. Therefore, glucose load plus intravenous insulin injection method was adopted to regulate glucose before GMMI. This method has been clinically proven to be a simple and effective glucoregulation regimen and recommended by ASNC and SNMMI^25^.

## Conclusions

No matter in the normal group or MI group, BW and SD values derived with GMMI phase analysis were in good agreements with those derived with GMPI. Especially in the MI group, both had a significant positive correlation (r_BW_ = 0.74, r_SD_ = 0.84). In addition, BW and SD of the normal group were significantly affected by the quality of PET images. After MI, left ventricle had evident systolic dyssynchony. Both BW and SD values were affected by multiple factors including TPD, cardiac function (ESV, LVEF and SMS), myocardial scar burden, and number of viable myocardial segments, indicating that LV systolic dyssynchrony after MI was the result of multi-factor interaction. Overall, GMMI phase analysis could accurately assess LV systolic dyssynchrony and could be promoted in clinics under assurance of image quality.

## Methods

### Experimental animals and grouping

A total of 18 adult healthy male Bama mini-pigs at age of 6 months old from the Academy of Agricultural Sciences, Jiangsu Province, China, were randomly divided into the normal group and MI group.

Based on our preliminary experiments, LV function and the degree of LV remodeling in pigs with MI change in a dynamic process and are in a stable state at 4 weeks after MI. Therefore, pigs in the MI group were subjected to 12-lead electrocardiogram, ^99m^Tc-methoxyisobutylisonitrile (MIBI) GMPI and ^18^F-FDG GMMI at 30–35 days after modeling. Upon the completion of imaging, pigs in the MI group, but not in the normal group, were sacrificed under anesthetic state for histopathological examination. The study was conducted in accordance with the Declaration of Helsinki and with the guide for care and use of laboratory animals. The experiment was approved by the Animal Ethics Committee of Soochow University and all efforts were made to minimize the suffering of animals. All methods were carried out in accordance with the approved protocols.

### MI model

To establish an animal model of MI, pigs in the MI group were deprived of water and feed for 12 h, intramuscularly injected 35 mg/kg ketamine and 1.5 mg/kg diazepam to induce anesthesia and 4–6 ml/h propofol to maintain anesthesia, and intravenously perfused 75 mg/h amiodarone to prevent malignant ventricular arrhythmia. The modeling was conducted under anesthesia as the following: (1) Perform tracheal intubation, connected pig to a ventilator to assist respiration, and subject pig to electrocardiogram and blood pressure monitoring throughout the operation; (2) MI was induced by catheter based 90-minute balloon occlusion of the LAD immediately after the origin of the first diagonal branch; and (3) Perform postoperative analgesia and anti-infection treatment. The successful establishment of pigs with MI was confirmed using electrocardiogram and the level of hypersensitive troponin I.

### ^99m^Tc-MIBI GMPI

At 30–35 days after modeling, the pigs in the normal and MI groups were deprived of water and feed for 12 h and subjected to GMPI using a Siemens Symbia T16 SPECT/CT Scanner equipped with a low-energy, high-resolution collimator (Germany). The procedure of GMPI was as follows: (1) Intravenously inject 370–555 MBq of ^99m^Tc-MIBI with radiochemical purity >95%; (2) 45 min after injection, fix pig and place electrodes upon the heart with two detectors setting at 90°; (3) Acquire images every 35 s and 6° clockwise from 45° at the right anterior oblique position to 45° at the left anterior oblique position with acquisition matrix of 128 × 128 and magnification of 1.45; (4) Perform the gated acquisition with electrocardiographic R-wave as the acquisition trigger signal and 8 frames/RR interval; and (5) Reconstruct images using filtered back projection method with cutoff frequency of 0.35 and order of 5 to obtain heart images in short axis, horizontal long axis and vertical long axis.

### ^18^F-FDG GMMI

GMMI was performed after GMPI at the same day using Siemens Biograph mCT-s (64) PET/CT Scanner (Germany). The procedure of GMMI was as follows: (1) Measure fasting blood glucose of each pig; (2) Intravenously inject 20–30 ml of 50% glucose solution (10–15 g glucose); (3) 30 min after injection, re-measure blood glucose level, (4) Intravenously inject insulin based on the glucose level according to the 2016 ASNC imaging guidelines/SNMMI procedure standard for PET nuclear cardiology procedures^25^ to adjust blood glucose to 5.55–7.77 mmol/l, 30 min later, re-measure blood glucose; (5) Intravenously inject 111–185 MBq of ^18^F-FDG; (6) At 45–60 min after injection, perform GMMI with 8 images per R-R interval, 3D acquisition, and 10 min/bed position; (7) Reconstruct images using algorithm of ultra high definition PET with 2 iterations and 21 subsets to obtain heart images in short axis, horizontal long axis and vertical long axis; and (8) Apply the Syngo TureD software to extract the ROI of myocardium and mediastinal blood pool, obtain their standard uptake value (SUVmyo and SUVbp), and calculate their ratio SUVm/b = SUVmyo/SUVbp.

### Phase analysis and assessment of the latest activation site of left ventricle

The reconstructed GMMI and GMPI images were subjected to phase analysis using Cedars QGS software (Cedars-Sinai Medical Center, Los Angeles, CA, USA) to obtain LV systolic synchronization parameters including phase BW and SD. Phase BW is defined as the range of degree of the cardiac cycle that encompasses 95% of the phase distribution, and phase SD is defined as the SD of the phase distribution. According to the initial phase angles of various LV myocardial segments, the segment with the maximum initial angle was defined as the latest activation site and its corresponding positions in LV anterior, anteroseptal, inferoseptal, inferior, inferolateral, anterolateral and apical region were recorded^15^.

### LV function analysis

GMPI images were also analyzed using software, Cedars QPS, to determine TPD, and GMMI images were analyzed using software, Cedars QGS, to obtain the entire LV functional parameters including EDV, ESV, and LVEF and LV regional functional parameters including SMS and STS.

### Assessment of viable and scar myocardium

The reconstructed short-axis GMMI images were displayed in the polar map format after normalized to the maximum activity and analyzed using a 17-segment model. Tracer uptake was quantitatively analyzed and categorized on a 4-point scale^4,22^: Point 0 was defined as tracer activity >75% (normal, viable); Point 1 as tracer activity = 50–75% (minimal scar); Point 2 as tracer activity = 25–50% (moderate scar); and Point 3 as tracer activity <25% (extensive scar). The number of viable (normal, score 0) segments per pig was noted. In addition, the summation of various segmental scores yielded the total scar score. The higher the score was, the more the scar tissue existed.

### Histopathology and staining

Upon the completion of imaging, the pigs in the MI group, but not in the normal group, were sacrificed under anesthetic state for histopathological examination using the following procedure: (1) Remove the heart immediately and rinse with saline; (2) Evenly cut the left ventricle into 6–8 pieces from the apex to the base, immerse these pieces in TTC at 37 °C for 10 min and subject them to pathological staining; (3) cut off tissues at infarct segment, infarct margin segment and normal myocardial segment, fix them in 10% formalin, embed them with paraffin and prepare as 5 μm sections; and (4) Stain these sections with HE and Masson so as to confirm the successful modeling and observe morphological changes in myocardial tissues.

### Statistical analysis

Statistical analysis was performed using SPSS 23.0 software and two-sided test. All count data were expressed as ratio and compared using chi-square test or Fisher exact probability method. Shapiro-Wilk test was performed to determine whether the measurement data follow a normal distribution. The measurement data were presented as mean ± standard deviation and compared using Mann-Whitney U test to examine the synchronization between MI and normal groups. Differences in LV systolic dyssynchrony of GMMI and GMPI were examined by comparing BW and SD using Wilcoxon symbol rank test. The correlation between their absolute value was analyzed by Pearson linear correlation analysis and Bland-Altman analysis. Intraclass correlation coefficient with absolute agreement definition was used to assess the degree of agreement between the two imaging methods. Multivariate stepwise regression analysis was used to analyze independent factors affecting BW and SD in GMMI. *P* < 0.05 was considered as statistically significant.
